# Supplementary material for: Differences in the proteomic profiles of the eutopic endometrium in patients with internal and external adenomyosis
Source: PLOS Glob Public Health. 2025 Jun 18;5(6):e0004785. doi: 10.1371/journal.pgph.0004785 (PMC12176133; doi:10.1371/journal.pgph.0004785)
Supplement: S1 Table — (DOCX) [file pgph.0004785.s001.docx]

**Supporting information S1. Sonographic findings**

| ID | Adenomyosis | Adenomyosis phenotype | Deep endometriosis (DE) | DE torus and/or uterosacral ligaments | DE recto-sigmoid | DE bladder | Ovarian endometrioma | Number or adenomyosis criteria | Wall asimmetry | Myometrial cysts | Hyperechogenic islands | Fan-shaped shadowing | Irregular or interrupted Junctional Zone | Translesional vascularity | Globular uterus |
| --- | --- | --- | --- | --- | --- | --- | --- | --- | --- | --- | --- | --- | --- | --- | --- |
| p1 | no | no | no | no | no | no | no | 0 | no | no | no | no | no | no | no |
| p3 | no | no | no | no | no | no | no | 0 | no | no | no | no | no | no | no |
| p11 | no | no | no | no | no | no | no | 0 | no | no | no | no | no | no | no |
| p12 | no | no | no | no | no | no | no | 0 | no | no | no | no | no | no | no |
| p13 | no | no | no | no | no | no | no | 0 | no | no | no | no | no | no | no |
| p14 | no | no | no | no | no | no | no | 0 | no | no | no | no | no | no | no |
| p15 | no | no | no | no | no | no | no | 0 | no | no | no | no | no | no | no |
| p16 | no | no | no | no | no | no | no | 0 | no | no | no | no | no | no | no |
| p25 | no | no | no | no | no | no | no | 0 | no | no | no | no | no | no | no |
| p31 | no | no | no | no | no | no | no | 0 | no | no | no | no | no | no | no |
| p32 | no | no | no | no | no | no | no | 0 | no | no | no | no | no | no | no |
| p35 | no | no | no | no | no | no | no | 0 | no | no | no | no | no | no | no |
| p36 | no | no | no | no | no | no | no | 0 | no | no | no | no | no | no | no |
| p38 | no | no | no | no | no | no | no | 0 | no | no | no | no | no | no | no |
| p39 | no | no | no | no | no | no | no | 0 | no | no | no | no | no | no | no |
| p40 | no | no | no | no | no | no | no | 0 | no | no | no | no | no | no | no |
| p46 | no | no | no | no | no | no | no | 0 | no | no | no | no | no | no | no |
| p101 | no | no | no | no | no | no | no | 0 | no | no | no | no | no | no | no |
| p56 | yes | internal | yes | yes | no | no | no | 4 | no | yes | yes | yes | yes | no | no |
| p57 | yes | internal | yes | yes | yes | yes | no | 5 | no | yes | yes | yes | yes | no | yes |
| p58 | yes | internal | yes | yes | yes | no | no | 5 | yes | yes | yes | yes | yes | no | no |
| p71 | yes | internal | yes | yes | yes | no | no | 4 | yes | yes | yes | no | yes | no | yes |
| p84 | yes | internal | no | no | no | no | yes | 6 | yes | yes | yes | yes | yes | no | yes |
| p87 | yes | internal | yes | yes | no | no | no | 7 | yes | yes | yes | yes | yes | yes | yes |
| p88 | yes | internal | yes | yes | no | no | yes | 7 | yes | yes | yes | yes | yes | yes | yes |
| p92 | yes | internal | yes | yes | yes | no | no | 7 | yes | yes | yes | yes | yes | yes | yes |
| p98 | yes | internal | yes | yes | no | no | yes | 6 | yes | yes | yes | yes | yes | no | yes |
| p52 | yes | external | yes | yes | yes | no | yes | 3 | yes | no | yes | yes | no | no | no |
| p54 | yes | external | yes | yes | yes | no | no | 3 | yes | no | yes | yes | no | no | yes |
| p65 | yes | external | yes | yes | no | no | no | 4 | no | yes | yes | yes | no | no | yes |
| p66 | yes | external | yes | yes | yes | no | yes | 3 | no | yes | yes | yes | no | no | no |
| p90 | yes | external | yes | yes | yes | no | no | 4 | no | yes | yes | yes | no | yes | no |
| p91 | yes | external | yes | yes | no | no | no | 4 | no | no | yes | yes | no | yes | yes |
| p95 | yes | external | yes | yes | no | no | yes | 3 | no | no | yes | yes | no | yes | no |
| p97 | yes | external | yes | yes | yes | no | no | 4 | yes | no | yes | yes | no | yes | no |
| p99 | yes | external | yes | yes | yes | no | yes | 3 | no | no | yes | yes | no | yes | no |
