# Supplementary material for: Differences in the proteomic profiles of the eutopic endometrium in patients with internal and external adenomyosis
Source: PLOS Glob Public Health. 2025 Jun 18;5(6):e0004785. doi: 10.1371/journal.pgph.0004785 (PMC12176133; doi:10.1371/journal.pgph.0004785)
Supplement: Attachment — Submitted filename: Response to reviewers.docx [file pgph.0004785.s006.docx]

**PGPH-D-24-02496
DIFFERENCES IN THE PROTEOMIC PROFILES OF THE EUTOPIC ENDOMETRIUM IN PATIENTS WITH INTERNAL AND EXTERNAL ADENOMYOSIS
PLOS Global Public Health**

**Response to Journal Requirements:**

**1. We noticed you have some minor occurrence of overlapping text with the following previous publication(s), which needs to be addressed:**

**https://pubmed.ncbi.nlm.nih.gov/38661227/**

**In your revision ensure you cite all your sources (including your own works), and quote or rephrase any duplicated text outside the methods section. Further consideration is dependent on these concerns being addressed.**

**Thank you for the recommendation, we made some changes, rephrasing the duplicated parts of the text we found.**

Abstract (l.24): “Two separate phenotypes of adenomyosis have been recognized, determined by the anatomical position of the adenomyotic lesions within the myometrium. This suggests that adenomyosis impacting the inner myometrium and that affecting the outer myometrial layer may have distinct origins and display different clinical and radiological characteristics.”

Methods (l.114): “For the ultrasound evaluation, we used the same methodology described in our previously published works(Valdés-Bango, Gracia, et al., 2024; Valdés-Bango, Ros, et al., 2024)”

**2. Please provide a/amend your detailed Financial Disclosure statement. This is published with the article. It must therefore be completed in full sentences and contain the exact wording you wish to be published.**

****Please only choose the relevant sentences from below****

**1. Please clarify all sources of funding (financial or material support) for your study. List the grants (with grant number) or organizations (with url) that supported your study, including funding received from your institution. 
2. State the initials, alongside each funding source, of each author to receive each grant.
3. State what role the funders took in the study. If the funders had no role in your study, please state: “The funders had no role in study design, data collection and analysis, decision to publish, or preparation of the manuscript.”
4. If any authors received a salary from any of your funders, please state which authors and which funders.**

**If you did not receive any funding for this study, please simply state: “The authors received no specific funding for this work.”**

Thank you for the recommendation. As requested, we have included the detailed Financial Disclosure statement below:

“This study has been funded by *Premio fin de residencia Emili Letang 2021* by Hospital Clínic de Barcelona received by M.V-B. (https://www.clinicbarcelona.org/en/docencia/residentes/post-residency-contracts). I confirm that the funder had no role in study design, data collection and analysis, decision to publish, or preparation of the manuscript.”

**3. Your current Financial Disclosure states, “The study was funded by the scholarship “Emily Letang”—Hospital Clinic of Barcelona, granted in May 2021.”. However, your funding information on the submission form indicates is missing. Please indicate by return email the full and correct funding information for your study and confirm the order in which funding contributions should appear. Please be sure to indicate whether the funders played any role in the study design, data collection and analysis, decision to publish, or preparation of the manuscript.**

This study has been funded by *Premio fin de residencia Emili Letang 2021* by Hospital Clínic de Barcelona received by M.V-B. (https://www.clinicbarcelona.org/en/docencia/residentes/post-residency-contracts). I confirm that the funder had no role in study design, data collection and analysis, decision to publish, or preparation of the manuscript.

**4. Thank you for uploading your study's underlying data set. Unfortunately, the repository you have noted in your Data Availability statement does not qualify as an acceptable data repository according to PLOS's standards.
 
 At this time, please upload the minimal data set necessary to replicate your study's findings to a stable, public repository (such as figshare or Dryad) and provide us with the relevant URLs, DOIs, or accession numbers that may be used to access these data. For a list of recommended repositories and additional information on PLOS standards for data deposition, please see** [**https://journals.plos.org/plosone/s/recommended-repositories**](https://journals.plos.org/plosone/s/recommended-repositories)**.**

Our ProtomeXchange dataset has been uploaded and been made public via PRIDE database.

Below are details:

**ProteomeXchange title:** Proteomic analysis of the eutopic endometrium of patients with internal or external adenomyosis

**ProteomeXchange accession:** PXD055131

**PubMed ID:** Not applicable

**Publication DOI:** Not applicable

**Project Webpage:**<http://www.ebi.ac.uk/pride/archive/projects/PXD055131>

**FTP Download:**<https://ftp.pride.ebi.ac.uk/pride/data/archive/2025/01/PXD055131>

The dataset was made public recently (today). We believe that, at the time of our first submission, it was not accessible without a username/password. In case it is still not publicly available, we have included the necessary information to access it below.

Reviewer account details:

**Username:** [reviewer_pxd055131@ebi.ac.uk](mailto:reviewer_pxd055131@ebi.ac.uk)

**Password:** 02t59mpovHmX

Don't forget to include these reviewer account details in your manuscript, during the peer review process.

Thank you for choosing PRIDE and ProteomeXchange for the dissemination of your data, and look forward to receiving more of your data in the future.

**5. Please insert an Ethics Statement at the beginning of your Methods section, under a subheading 'Ethics Statement'. It must include:**

**1) The name(s) of the Institutional Review Board(s) or Ethics Committee(s)**

**2) The approval number(s), or a statement that approval was granted by the named board(s)**

**3) (for human participants/donors) - A statement that formal consent was obtained (must state whether verbal/written) OR the reason consent was not obtained (e.g. anonymity). NOTE: If child participants, the statement must declare that formal consent was obtained from the parent/guardian.**

Thank you for the comment. We inserted an Ethics Statement at the beginning of the Methods section as requested (l.99): **“Ethics Statement:** Ethical approval was obtained from the Ethics Committee for Biomedical Research - Hospital Clínic de Barcelona (Approval Number: HCB/2021/0242). All the study participants provided written informed consent before the study started.”

**6. Please provide an Author Summary. This should appear in your manuscript between the Abstract (if applicable) and the Introduction, and should be 150–200 words long. The aim should be to make your findings accessible to a wide audience that includes both scientists and non-scientists. Sample summaries can be found on our website under Submission Guidelines:<https://journals.plos.org/globalpublichealth/s/submission-guidelines#loc-parts-of-a-submission>**

We added an Authors Summary as requested (l.49): “In this study, we explored a gynecological condition called adenomyosis, where tissue from the inner lining of the uterus grows into the muscular wall. Adenomyosis can affect different areas of the uterine muscle, and we know there are two distinct forms: one that involves the inner layer of the muscle and another that affects the outer layer. Our aim was to understand how these two forms of adenomyosis might differ at a molecular level by looking at the proteins present in the endometrium (the inner lining of the uterus). We found significant differences in the protein profiles between women with the two forms of adenomyosis and healthy women. In those with the external form, there was a stronger immune response and more signs of inflammation. On the other hand, the internal form showed changes in proteins involved in cell movement and cell death. These findings suggest that the two forms of adenomyosis may have different causes and biological processes. This could lead to more targeted treatments in the future, and our study highlights the importance of further research to understand these differences and their impact on patient care.”

**7. We have noticed that you have a list of Supporting Information legends in your manuscript. However, the Supplementary File 1's label and caption is written as "Supplementary file 2. Sonographic findings". Please upload them as separate files with the item type 'Supporting Information'.** 

We changed the name of the files from Supplementary File…. to Supporting Information with the correct number Supporting Information S1, Supporting Information S2….

Additional Editor Comments (if provided):

[Note: HTML markup is below. Please do not edit.

**Reviewer's Responses to Questions**

**Comments to the Author**

1. Does this manuscript meet PLOS Global Public Health’s [publication criteria](https://journals.plos.org/globalpublichealth/s/criteria-for-publication)? Is the manuscript technically sound, and do the data support the conclusions? The manuscript must describe methodologically and ethically rigorous research with conclusions that are appropriately drawn based on the data presented.

Reviewer #1: Yes

2. Has the statistical analysis been performed appropriately and rigorously?

Reviewer #1: Yes

3. Have the authors made all data underlying the findings in their manuscript fully available (please refer to the Data Availability Statement at the start of the manuscript PDF file)?

The [PLOS Data policy](https://journals.plos.org/globalpublichealth/s/data-availability) requires authors to make all data underlying the findings described in their manuscript fully available without restriction, with rare exception. The data should be provided as part of the manuscript or its supporting information, or deposited to a public repository. For example, in addition to summary statistics, the data points behind means, medians and variance measures should be available. If there are restrictions on publicly sharing data—e.g. participant privacy or use of data from a third party—those must be specified.

Reviewer #1: Yes

4. Is the manuscript presented in an intelligible fashion and written in standard English?

PLOS Global Public Health does not copyedit accepted manuscripts, so the language in submitted articles must be clear, correct, and unambiguous. Any typographical or grammatical errors should be corrected at revision, so please note any specific errors here.

Reviewer #1: Yes

5. Review Comments to the Author

Please use the space provided to explain your answers to the questions above. You may also include additional comments for the author, including concerns about dual publication, research ethics, or publication ethics. (Please upload your review as an attachment if it exceeds 20,000 characters)

Reviewer #1: The study aims to investigate the proteomic profiles of different adenomyosis phenotypes, which is a well-defined and relevant objective given the clinical implications of adenomyosis. I have some comments listed below to be answered before the consideration of publication.

**1. Regarding the sonographic findings among different groups, all patients in the internal In group have irregular or interrupted junctional zone, while all the patients in the external group are not. In general, the thickness of the junctional zone over 12 mm is in favor of adenomyosis, and abnormal junctional zone are thought to involve in the pathogenesis of adenomyosis. Have the authors considered to measure and compare the thickness of junctional zone in internal and external groups? Do authors think there will be a difference regarding the thickness of junctional zone between internal and external adenomyosis?**

Indeed, as mentioned by the revisor, all patients in the internal adenomyosis group had irregular or interrupted junctional zone, while all the patients in the external group had not. This is due to the definitions of internal and external adenomyosis themselves. As we mentioned in the introduction, “To distinguish between internal and external adenomyosis we employed the following sonographic categorization of the myometrium into three layers: the inner myometrium (corresponding to the JZ), the middle myometrium extending from the JZ to the venous and arterial arcuate vessels of the uterus, and the outer myometrium located between the arcuate vessels and the uterine serosa***.*** The vascular arcuate, observed in the sagittal plane in two-dimensional transvaginal ultrasound (2D TVUS) with the application of color or power Doppler, served as a reference for the differentiation of uterine layers (Van den Bosch et al., 2019). **Internal adenomyosis was defined when the JZ (inner myometrium) was affected** with or without involvement of the middle myometrium and without affecting the outer myometrium (subserosal layer). **External adenomyosis, on the other hand, was considered when it involved the outer myometrium**, with or without involvement of the middle myometrium, **without altering the JZ**.

As noted by the reviewer, a junctional zone thickness greater than 12 mm is a widely used radiological criterion, assessed by MRI, to diagnose adenomyosis (Rees et al., 2021). Furthermore, molecular abnormalities in the junctional zone are believed to be involved in the pathogenesis of adenomyosis (García-Solares et al., 2018).

However, while the measurement of junctional zone thickness by ultrasound (both 2D and 3D) was considered in the past, it was deemed non-reproducible and controversial. In the latest consensus on the revised MUSA criteria for adenomyosis following the 2022 Delphi procedure, experts concluded that ultrasound measurement of junctional zone thickness has no current role in clinical practice. Regarding the evaluation of the junctional zone, the consensus stated: " They [the experts] suggested assessing the regularity of the junctional zone in multiple planes using 3D ultrasound (any plane slicing). The suggestion to measure the maximal thickness of the junctional zone, based on responses from the first round (Table 1), was dismissed by the experts due to a lack of evidence supporting the clinical relevance of this measurement” (Harmsen et al., 2022).

For these reasons, we did not consider measuring and comparing the junctional zone thickness between patients with internal and external adenomyosis, but instead focused on evaluating the irregularity or interruption, as defined by MUSA criteria. However, as the reviewer suggested, if we had measured it, we likely would have found differences between the groups, with the internal adenomyosis group showing a thicker junctional zone. We could also hypothesize that the etiopathogenic mechanisms at the junctional zone level, such as endometrial invagination, tissue injury and repair, and epithelial-mesenchymal transition (García-Solares et al., 2018),are more strongly associated with the internal phenotype of the disease and have a radiological correlate.

**2. The authors mentioned that this is the first study comparing external and internal adenomyosis at a proteomic level and the findings support distinct pathogenic mechanisms for the two phenotypes. Based on the proteomic findings in the external adenomyosis, how will the authors explain the pathogenesis of external adenomyosis and correlate the findings with symptoms in external adenomyosis? What is the biggest difference regarding the pathogenic mechanism compared to internal adenomyosis?**

While the exact mechanisms underlying the development of adenomyosis remain unclear, histopathological evidence suggests that adenomyosis affecting the inner myometrium may arise from the invasion of endometrial tissue through the myometrium or from de novo metaplasia of embryonic or adult stem cells within the myometrium. In contrast, adenomyosis involving the external myometrial layer is thought to develop through the invasion of ectopic endometrial cells from adjacent endometriotic lesions or by the differentiation of adult endometrial and stromal stem cells deposited in the myometrium due to retrograde menstruation.

Our study identified key immune-related differences between external and internal adenomyosis, with external adenomyosis exhibiting notably more active immune pathways, including complement activation, phagocytosis, and neutrophil involvement, compared to the internal phenotype. These findings are consistent with similar immune alterations observed in endometriosis, such as increased neutrophil activity and elevated IL-8 levels, which facilitate neutrophil recruitment and inflammation. Neutrophils are also involved in promoting angiogenesis and immune modulation, while cytokines such as TGFB1, IL4, and IFNG contribute to inflammation, tissue remodeling, and fibrosis in both adenomyosis and endometriosis. Furthermore, the complement system’s role in recognizing pathogens and modulating the immune response may play a crucial part in the inflammatory processes seen in external adenomyosis.

These immune-related findings align with clinical features of external adenomyosis described in previous studies (Bourdon et al., 2021; Exacoustos et al., 2020; Kishi et al., 2017; Valdés-Bango et al., 2024). In a study published by our team (Valdés-Bango et al., 2024), we found that external adenomyosis is independently associated with the presence of deep infiltrating endometriosis (DE) and nulliparity, both of which support the theory of an ectopic endometrial origin, potentially exacerbated by retrograde menstruation and inflammatory processes. In contrast, internal adenomyosis, which shows less immune activation, was strongly associated with leiomyomas. This suggests that the internal phenotype may arise from endometrial invasion or stem cell metaplasia within the myometrium, driven by dysregulation of several cellular functions, such as the cell cycle, cell death, and invasiveness. These combined immune and clinical findings provide valuable insights into the distinct pathogenic mechanisms of the two adenomyosis phenotypes, highlighting the potential for tailored diagnostic and therapeutic approaches.

We added in the manuscript (l.350-355): “In a previous study published by our team (Valdés-Bango et al., 2024), we found that external adenomyosis is independently associated with the presence of deep infiltrating endometriosis (DE) and nulliparity, both of which support the theory of an ectopic endometrial origin, potentially exacerbated by retrograde menstruation and inflammatory processes. In contrast, internal adenomyosis, was strongly associated with leiomyomas.”

Some minor suggestions:

**3. Do authors miss internal in the title of Table 2 and 3?
- Table 2. Top 25 activated upstream regulators in external vs xx adenomyosis
- Table 3. Top 25 inhibited upstream regulators in external vs xx adenomyosis**

Indeed, thank you for your comment. We corrected the titles of the tables to:

- Table 2. Top 25 activated upstream regulators in external vs internal adenomyosis
- Table 3. Top 25 inhibited upstream regulators in external vs internal adenomyosis.

**4. In Figure 4, the threshold line is too thin to recognize, especially the figure 4a, considering the bar color is orange, the orange threshold line is too vague.**

Thank you for your comment, we made a change in the figure as suggested a clearer design.

**5. In Figure 3, it seems a bracket is missing in the Y axis. -log p value should be -log(p-value)**

Thank you for your comment, we change it as suggested.

**6. In supplementary file 1, there is typo about '' interrumped '' should be ''interrupted''**

Thank you for your comment, we made the correction from “interrumped” to interrupted” in the supporting information S1 file.

Overall, this study provides valuable insights into the distinct proteomic profile of internal and external adenomyosis, laying the groundwork for future research and potential diagnostic and therapeutic advancements.

6. PLOS authors have the option to publish the peer review history of their article ([what does this mean?](https://journals.plos.org/globalpublichealth/s/editorial-and-peer-review-process#loc-peer-review-history)). If published, this will include your full peer review and any attached files.


**Do you want your identity to be public for this peer review?** If you choose “no”, your identity will remain anonymous but your review may still be made public.


For information about this choice, including consent withdrawal, please see our [Privacy Policy](https://www.plos.org/privacy-policy).

Reviewer #1: No

[NOTE: If reviewer comments were submitted as an attachment file, they will be attached to this email and accessible via the submission site. Please log into your account, locate the manuscript record, and check for the action link "View Attachments". If this link does not appear, there are no attachment files.]

While revising your submission, please upload your figure files to the Preflight Analysis and Conversion Engine (PACE) digital diagnostic tool, https://pacev2.apexcovantage.com/. PACE helps ensure that figures meet PLOS requirements. To use PACE, you must first register as a user. Registration is free. Then, login and navigate to the UPLOAD tab, where you will find detailed instructions on how to use the tool. If you encounter any issues or have any questions when using PACE, please email PLOS at figures@plos.org. Please note that Supporting Information files do not need this step.
