# Supplementary material for: Differences in the proteomic profiles of the eutopic endometrium in patients with internal and external adenomyosis
Source: PLOS Glob Public Health. 2025 Jun 18;5(6):e0004785. doi: 10.1371/journal.pgph.0004785 (PMC12176133; doi:10.1371/journal.pgph.0004785)
Supplement: Attachment — Submitted filename: Response to reviewers_1903.docx [file pgph.0004785.s007.docx]

**PGPH-D-24-02496R1
DIFFERENCES IN THE PROTEOMIC PROFILES OF THE EUTOPIC ENDOMETRIUM IN PATIENTS WITH INTERNAL AND EXTERNAL ADENOMYOSIS
PLOS Global Public Health


Response to Journal Requirements:**

**1. Please review your reference list to ensure that it is complete and correct. If you have cited papers that have been retracted, please include the rationale for doing so in the manuscript text or remove these references and replace them with relevant current references. Any changes to the reference list should be mentioned in the rebuttal letter that accompanies your revised manuscript. If you need to cite a retracted article, indicate the article’s retracted status in the References list and also include a citation and full reference for the retraction notice.**

Thank you for your comment. We have thoroughly reviewed the reference list and corrected any errors related to repeated references. Additionally, we have checked that none of the included references have been retracted. If any changes were made to the reference list, we have addressed them in the accompanying rebuttal letter.

**Responses to Reviewers’ Comments and Questions:**

6. Review Comments to the Author

Please use the space provided to explain your answers to the questions above. You may also include additional comments for the author, including concerns about dual publication, research ethics, or publication ethics. (Please upload your review as an attachment if it exceeds 20,000 characters)

**Reviewer #1:**

**This manuscript makes a contribution by pioneering the comparative proteomic analysis of endometrial tissues between internal and external adenomyosis phenotypes. The authors appropriately acknowledge limitations, including dynamic range constraints and sample size. Their suggestion to include adenomyotic lesions and myometrial samples in future research demonstrates forward thinking. This work establishes a foundation for targeted diagnostic and therapeutic approaches for adenomyosis subtypes, making it worthy of publication.

Some small corrections and points to reduce the redundancy of the text are listed below.

-Line 286 ‘dysfunction in the inflammatory response´, ‘the’ is missing.**

Thank you for the comment. We corrected the sentence adding the missing “the” (l.291).

**-Line 289-291 ´When focusing on the comparison between external and internal adenomyosis phenotypes, our results revealed that the eutopic endometrium from women with internal and external adenomyosis exhibits significantly different proteomic profiles´. The sentence is too redundant and could be simplified and shortened as the example, ´ The eutopic endometrium from women with internal versus external adenomyosis shows significantly different protein profiles´**

Thank you for the comment. We changed the sentence as suggested to make it clearer and less redundant (l.294-295): “The eutopic endometrium from women with internal versus external adenomyosis shows significantly different proteomic profiles.”

**-Line 336 ´common to both phenotypes’ the preposition should be ‘in’**

Thank you for the comment. We changed the preposition as suggested (l.342).

**Reviewer #2:**

**- Please, carefully revised the references. Ref 15 and 48 are exactly the same paper. If the same first author and same year of publication, put a and b to differentiate them. Also, be consistent and report all references in the same way (using numbers). Don't use author's names like lines 71, 82, and 89.**

Thank you for your comment. We have thoroughly reviewed the reference list and corrected any errors related to repeated references. Additionally, we have made the necessary changes to ensure consistency in citation style, and all references are now reported in the same way using numbers.

**- Line 78: add a reference to this sentence 'it is known that patients with adenomyosis present an immune dysregulation leading to chronic inflammation as well as imbalances in steroidal hormones'**

We added the corresponding reference as suggested (l.77): “(14)”.

**- Line 89: Among transcriptomic studies, you may want to mention this paper that just came out:
 Zipponi M. et al. Endometrial stromal cell signaling and microRNA exosome content in women with adenomyosis. Mol Hum Reprod. 2025 Jan 17;31(1):gaae044. doi: 10.1093/molehr/gaae044.
They found that miR-1 was downregulated in adenomyosis. This information could be implemented in lines 333-334 to add value and quality to your results reported in table 3.I suggest to cite this paper in lines 327-328 when talking about ERK/MPAK enrichment in adenomyosis, as well as this very recent paper:
Jia, JJ., Lai, Hj., Sun, BW. et al. miR-21 regulates autophagy and apoptosis of ectopic endometrial stromal cells of adenomyosis via PI3K/ AKT/ mTOR pathway. Sci Rep 15, 7639 (2025). doi:** [**https://doi.org/10.1038/s41598-025-92526-3**](https://doi.org/10.1038/s41598-025-92526-3)**.**

Thank you for your comment. Indeed, these articles were not published at the time of the initial submission of our manuscript. As suggested, we have mentioned both recommended papers in the introduction (including both references (30) and (31) when referring to previous transcriptomic and proteomic studies in l.88) and in the discussion (l.332-335), adding value to our reported results: “Recently studies published by Zipponi et al. (30) and Jia et al. (31)also suggest that the dysregulation of multiple microRNAs can influence the progression of adenomyosis through the PI3K/AKT/mTOR pathway, which is involved in cell growth, survival, and proliferation.”

**- Line 151: for how long did you store fresh pellet at -80°C prior to analysis?**

We added the following information in the material and methods section to clarify it (l.147-148): “Pellets were stored for up to six months, from collection and initial processing, until all participant samples were collected, and final analysis conducted”.

**- Line 163: how did you quantify 200 ng? This information is missing. Please, revise this paragraph 'Mass spectrometry analysis' which is confusing. Did you quantify proteins before loading them into the liquid cromatograph or this type of information was retrieved after with DIA software? It is not clear at all.**

Materials and methods section has been modified according to reviewer’s instructions (l.154-156, 160, 166). Briefly, protein was quantified prior digestion, and then C18 stage tips were used to clean up the sample prior acquisition, but also to process a limited amount of sample (2µg). Then, a small portion of the processed sample (200ng) was loaded onto the timsTOF Pro. This is of paramount importance since the preliminary version of the tims cartridge saturates above 200ng peptide load. We hope the information is now clearer.

**- Line 304: add a reference to this sentence 'previous studies have described an increased abundance and activity of neutrophils in the eutopic proliferative endometrium of patients with endometriosis, as well as elevated levels of IL-8, which may enhance neutrophil recruitment'**

We added the corresponding reference (41) to the sentence in l.307 as requested.

**- Write the word 'p-value' in the same way in the manuscript and in the figures.**

Thank you for the comment. We made the corresponding changes in Figures 1 and 2, as well as in the manuscript. The term 'p-value' is now written consistently throughout the manuscript. **Figures:
Figure 1. The volcano plots should have the same scale for the x-axis.**

The Volcano plot-generating tool employed (SRplot) adjusts the display of the results to the range of the data under analysis. This improves the resolution of the data points and helps their interpretation. Regretfully, the software doesn’t allow the establishment of a fixed value in none of the axes.

**Figure 3. Define GO_BP in the legend as gene ontology_biological processes and explain the difference with the term signaling pathways. Why did you decide to look for biological pathways?**

The corresponding figure caption and body text have been modified according to reviewer’s instructions (l.239-240, l.242-247). The IPA software program uses both public and proprietary knowledge for a deeper and more precise characterization of the molecular events underlying the biological systems under analysis. In addition, the analysis of the protein expression fold change patterns adds an additional level of data interpretation through the Z-score. This parameter infers likely activation or inhibition states of the biological functions/pathways/upstream regulators based on the expression pattern afore mentioned.

**Figure 4. It is confusing. Both graphs goes from left to right even when showing -log(p-values)<0. I suggest to put results in un graph only. It is easier to understand that the right part shows upregulated pathways (>0) and the left part shows downregulated ones (<0). Also I would prefer to see these results expressed in z-score (X axis) like mentioned in materials&methods (line 185). Set the threshold line at 2 'Z‐score values ≥ 2 or <-2 were considered as significantly activated or inhibited'.**

As explained in the Materials and Methods section, IPA first performs an overlap analysis where it establishes whether there is a statistically significant overlap between the set of proteins regulated in our system (differentially expressed proteins) and the proteins/genes related to a specific pathway. This correlation is calculated by a Fisher’s exact test, and the results with a p<0.05 are considered as significant (more genes related to a certain pathway than expected by chance alone). However, these p values are graphed as -log p value, so that higher significance leads to a greater value. The cutoff, corresponding to p<0.05, is -log p value> 1.3. Therefore, all results displayed in the bars are positives.

Then, IPA calculates the Z score based on the predicted effect that the proteins display over the pathway itself, and viceversa. This leads to a theoretical inference of the activation or inhibition state of the pathway. The graphs display Z scores information using a color code: orange for Z scores>0 (theoretical activation of the pathway), and blue for Z scores<0 (theoretical inhibition of the pathway).

We modified figure 4. We believe the current display where both -log p values and Z scores are represented adds valuable information on the system under analysis, so we have rearranged the bar chart to clarify as much as possible the results shown.

Current figure 4 summarizes the overlap significance (-log p-value) of the top enriched pathways, but now we have selected only those results that pass the Z score threshold of >2 or <-2 for the consideration of a reliable inference of activation or inhibition, respectively (l.259-260). In addition, Z score values for each of the selected pathways are now shown inside the bars.
